# Implicit learning of unfamiliar tone sandhi patterns in lexical recognition

**DOI:** 10.3389/fpsyg.2024.1414732

**Published:** 2025-01-29

**Authors:** Ting Zou, Xinbing Luo

**Affiliations:** School of English and International Studies, Beijing Foreign Studies University, Beijing, China

**Keywords:** implicit learning, tone sandhi, adult L2 learners, attention, Tianjin Mandarin, artificial word learning

## Abstract

**Introduction:**

This study investigates whether unfamiliar tone sandhi patterns in Tianjin Mandarin can be implicitly learned through an artificial language learning experiment, and if the acquired knowledge is rule-based and generalizable.

**Methods:**

Participants were trained to learn monosyllabic words and disyllabic phrases with their attention focused on a word-order rule, while unknowingly being exposed to unfamiliar tone sandhi patterns. A judgement test with trial-to-trial confidence ratings was conducted to assess the learning outcomes and participants’ awareness.

**Results:**

Results revealed significantly above-chance performance on tone sandhi patterns for learned phrases. This learning effect was generalized to unseen phrases made up of familiar words, but not to phrases with new words, indicating a degree of abstraction across instances, though the learning is not fully rule-based. The confidence rating results suggest that participants were unaware of the structural sandhi knowledge, but the reaction time data of the judgement test indicate that the sandhi knowledge was learned with awareness at the level of noticing.

**Discussion:**

The results have been discussed in light of theories of implicit learning and the findings of previous research on phonological learning.

## 1 Introduction

Implicit learning refers to a natural process in which learners acquire underlying regularities in the environment without intention (Berry, 1997; Ellis, 2015; Reber, 1967). Contrary to the deliberate and reflective nature of explicit learning, implicit learning is automatic and unintentional since it always takes place in incidental conditions, with the learning process dissociated from awareness (see Cleeremans et al., 1998; Perruchet, 2008; Shanks, 2005; Stadler and Frensch, 1997, for reviews). For example, people could encode the occurrence frequency of events automatically (Zacks et al., 1982).

As a primary form of learning, implicit learning has been extensively studied for its cognitive role in the formation of skills and behaviours (e.g., Althoff and Cohen, 1999; Kihlstrom, 1987). Naturally, implicit learning is also crucial in extracting underlying regularities when we are “picking up” languages (e.g., Williams, 2009). Past research has demonstrated that the general ability of implicit learning is intrinsically associated with speakers’ language performance (Kaufman et al., 2010). More specifically, implicit learning has been exhibited to play a significant role in the acquisition of inflectional morphology (e.g., Brooks and Kempe, 2013), syntactic patterns (e.g., Rebuschat, 2009; Williams and Kuribara, 2008) and form-meaning mappings (e.g., Li et al., 2020; Paciorek and Williams, 2015). While previous studies on the implicit learning of phonological patterns have mainly focused on phonotactic rules and word stress patterns (see section 1.1 for details), there is a limited number of research on the learning of lexical tone variations. Notably, few studies have directly assessed participants’ awareness and the nature of the resultant knowledge. Therefore, the current study aims to shed further light on whether a phonological pattern from a natural language, i.e., tone sandhi patterns in Tianjin Mandarin, can be learned implicitly in a word recognition situation, and whether the resultant knowledge can be rule-based and generalizable.

### 1.1 The specificity of implicit learning in phonological rules

The specificity of the knowledge acquired and how that knowledge is represented remains a crucial issue in the realm of implicit learning. Some studies have proposed a rule-based account, suggesting that the tacit knowledge acquired without deliberate effort is abstract and generalizable (e.g., Reber, 1989). This perspective has received substantial empirical support, particularly in the context of artificial grammar learning (e.g., Brooks and Vokey, 1991; Gomez and Gerken, 1999; Goschke and Bolte, 2007; Scott and Dienes, 2010). Studies have shown that even infants are capable of rapidly representing and extracting algebra-like rules from limited amount of input, and generalizing such rules to novel items (Marcus et al., 1999). Additionally, previous findings have revealed that artificial grammar knowledge could be generalized from one modality (e.g., sequences of tones differing in pitch) to another modality (e.g., sequences of arbitrary graphical symbols; Altmann et al., 1995).

In contrast, other research on artificial grammar learning has bolstered the claim of an instance-based learning, suggesting that each learning experience leaves a distinct memory trace, and subsequent performance relies on the retrieval of one of the traces (e.g., Goldinger, 1998; Jamieson and Mewhort, 2011). Evidence from this perspective showed that the knowledge resulting from implicit learning is primarily based on memorization of specific chunks and particular exemplars (e.g., Jamieson and Mewhort, 2011; Perruchet and Pacteau, 1990).

With respect to the research on the implicit learning of phonological aspect of language, the learning of segmental phonotactic constraints has been extensively studied (Dell et al., 2000; Goldrick, 2004; Kittredge and Dell, 2016; Onishi et al., 2002; Warker et al., 2008; Chambers et al., 2010). These studies generally indicate that the acquired knowledge is rule-based and generalizable. For instance, in the study by Dell et al. (2000), participants were asked to read aloud artificial CVC syllables without being aware of the exact regularities governing the position of the segments. The results showed that the participants’ slips of the tongue reflected the phonotactic distributions they had been exposed to, and this pattern was also found in novel syllables that had not been practiced, indicating that participants learned more than just a syllable inventory; they acquired the underlying rules. A later study by Chambers et al.’ (2010) demonstrated that participants responded more quickly to constraint-following (legal) than to constraint-violating (illegal) syllables. This legality advantage in response latency could be rapidly transferred to novel vowel context, suggesting that the rule-based abstract phonotactic knowledge could be acquired.

In the realm of prosodic learning, Chan and Leung (2014) found that after a brief auditory exposure to novel words, Cantonese speakers could endorse the forms with correct word stress pattern significantly better than chance in an acceptability judgement test, even though they were unaware of the underlying Spanish stress assignment rules. Graham and Williams (2018) demonstrated that native Japanese listeners could implicitly learn unfamiliar stress pattern regularities after a brief auditory exposure. They further observed a “seamless generalization” of the newly acquired stress patterns to novel items, with no evident transfer decrement in participants’ performance. Chan and Leung (2018) confirmed this implicit learning by assessing participants’ awareness using both subjective measures (retrospective verbal reports) and objective measures (confidence ratings), showing that participants could indeed acquire abstract and potentially rule-based knowledge of word stress patterns.

The learning of lexical tone patterns has also garnered research attention (Chan and Leung, 2020; Chen, 2020, 2022; Huang and Do, 2021; Leung, 2004). For example, Leung (2004) demonstrated that attention plays a crucial role in successful implicit learning, as participants successfully acquired the grammar governing tonal sequences when attention was directed solely to tones, and this knowledge could be transferred to sequences consisting of novel syllables. Using an artificial language learning paradigm, Huang and Do (2021) tested the implicit learning of tonal alternations and found a learning bias toward a uni-directional, right-dominant tone deletion pattern, consistent with the directionality of tone sandhi patterns observed in many Chinese dialects. This asymmetry in tone deletion direction may reflect a phonetically grounded structural learning bias. Similarly, in an implicit artificial learning experiment, Chen (2020) uncovered an inductive learning bias favoring tonal phonotactics that forbid non-domain-final rising tones (*NonFinalR) over those that ban non-domain-final high-level tones (*NonFinalH). The inherent difficulty of f0 elevation—making pitch raising more challenging to achieve in non-domain-final positions—underpins this innate preference.

However, since the above-mentioned tone learning studies were mainly designed to test specific phonetic or phonological learning hypothesis, participants’ awareness in experiments claiming to probe implicit learning was not directly measured. As a result, it was not always clear whether the acquired knowledge was obtained without conscious intent (see, for example, Chan and Leung, 2018; Rebuschat, 2013). Moreover, the target tonal variation patterns in previous research were designed particularly for hypothesis testing, such as the tone deletion patterns in Huang and Do (2021), and the averaged and manipulated f0 contours in Chen (2000). These scenarios are clearly far removed from more natural language learning situations, such as second dialect acquisition setting. Therefore, it remains to be determined whether a tonal variation pattern existing in natural language, such as tone sandhi patterns in Tianjin Mandarin, can be acquired implicitly in word recognition situation, and whether participants can automatically detect patterns across instances, leading to potentially rule-based and generalizable knowledge.

### 1.2 Tone sandhi patterns in standard Chinese and Tianjin Mandarin

Tone sandhi is a widespread phenomenon in tone languages, referring to the alternations of lexical tones that triggered by specific linguistic context in connected speech (see Chen, 2000 for a review). When one tone-bearing syllable is juxtaposed to other tone-bearing syllables, its citation form (i.e., the tone when it is produced in isolation) may systematically deviate under certain conditions. For example, in Standard Chinese, when Tone 3 is followed by another Tone 3, the preceding one will change into a Tone 2-like rising tone, i.e., Tone 3 → [+Rising]/___Tone 3. (e.g., Chen, 2000; Duanmu, 2000). Studies on the representation of sandhi patterns have generally revealed that native speakers tend to represent disyllabic Tone 3 sandhi words in their underlying form (Tone 3 + Tone 3), and this representation involves an online computation mechanism (Chien et al., 2016; Meng et al., 2021).

As a variant of Mandarin, Tianjin Mandarin is spoken by residents in Tianjin, a city adjacent to Beijing. Like Beijing Mandarin, the tonal inventory of Tianjin Mandarin also consists of four lexical tones, namely Tone 1 (T1, a low-register falling tone), Tone 2 (T2, a high-register rising tone), Tone 3 (T3, a low-register dipping tone), and Tone 4 (T4, a high-register falling tone; Li and Chen, 2016).

In Tianjin Mandarin, tonal variations are abundant, and early studies generally recognized four sandhi patterns, namely (1) T1 + T1 (L + L) → T3 + T1 (LH + L); (2) T3 + T3 (LH + LH) → T2 + T3 (H + LH); (3) T4 + T1 (HL + L) → T2 + T1 (H + L); (4) T4 + T4 (HL + HL) → T1 + T4 (L + HL; e.g., Hung, 1987; Tan, 1987; Hyman, 2007). Among these patterns, Rule (4) has been argued to have become obsolete among younger generations, with only a small subset of elder speakers maintaining its use on a limited set of words (Zhang and Liu, 2016).

**Figure 1:**
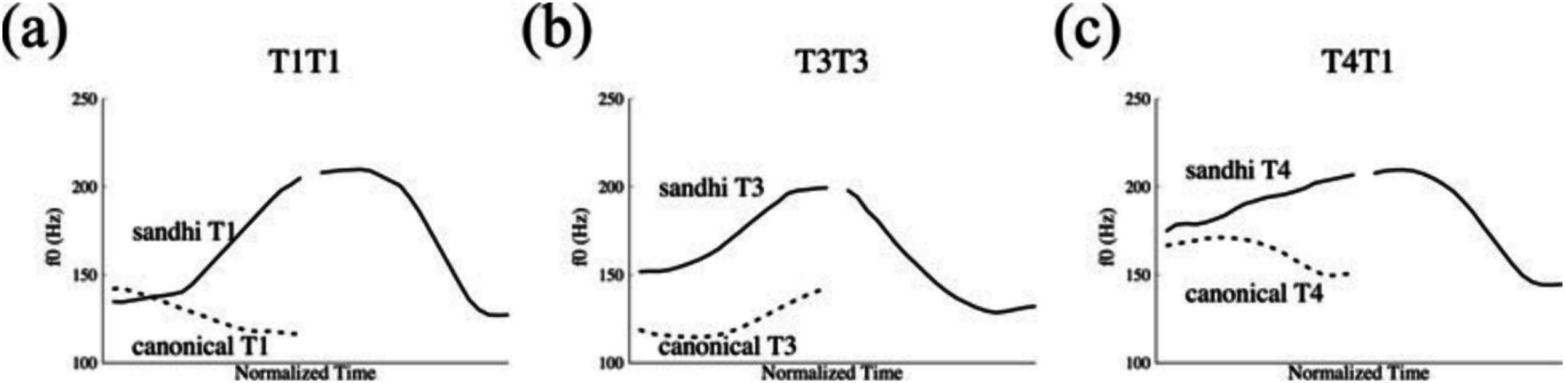
The F0 realizations of three tone sandhi patterns in Tianjin Mandarin with solid lines indicating the sandhi patterns and dashed lines for the canonical tonal contours (from Li and Chen, 2016). **(Panels A–C)** display the underlying and sandhi forms of T1T1, T3T3, and T4T1, respectively.

Recent empirical studies using fine-grained acoustic measurement of f0 contours (Li and Chen, 2016; Li et al., 2019) have confirmed the validity of the sandhi patterns in T1T1, T4T1 and T3T3, in which the sandhi tones present significant deviation from their canonical tone contours (as shown in Figure 1). However, contrary to earlier claims that these sandhi patterns involve categorical changes in tone, as stipulated in Rules (1–3), recent studies have revealed that when occurring before T1, T1 and T4 do not undergo a categorical shift to T3 and T2, respectively. Instead, in these contexts, T1 and T4 are simply realized with rising f0 contours that are distinct from their canonical tone forms. Therefore, this sandhi pattern can be summarized into a simplified phonological rule: {T1, T4} → [+Rising]/___T1. For native Tianjin Mandarin speakers, these sandhi patterns can be generalized and applied in novel words with various degree of productivity, as demonstrated by the results of a Wug test (Zhang and Liu, 2016).

Recently, several studies have further explored the productivity of sandhi patterns among L2 learners of Standard Chinese (e.g., Chen et al., 2019, 2022; Qin, 2022). For instance, Chen et al. (2019) observed that Cantonese and American learners of Standard Chinese could apply T3 sandhi rules consistently to both real words and nonsense words, indicating that sandhi forms are integrated into the mental representation of the abstract T3 category. Although L2 learners produced tone contours with less detail and precision compared to native speakers, the findings suggested that the computation of allophonic variants could take place in L2 Chinese production.

Likewise, implicit learning of tone sandhi patterns could naturally take place during the acquisition of a second dialect, particular when an individual relocates to a different region and gradually adapts to the local linguistic environment (Chang et al., 2012). Research has shown that the consonantal allophones from a dialect could be acquired during brief conversations without conscious awareness (e.g., German et al., 2013). Therefore, the current study sought to investigate whether the T1T1 and T1T4 sandhi patterns in Tianjin Mandarin could be learned in an implicit manner by native Standard Mandarin speakers from Beijing in an artificial language learning experiment.

### 1.3 Gauging participants’ awareness in the learning process

In order to assess the implicitness of the acquired knowledge, measurements such as verbal report and confidence rating were used in previous research. Retrospective verbal reports, a widely used method, involve participants verbalizing any patterns they noticed after the test (Williams, 2005; Rebuschat, 2013; Rebuschat et al., 2015). If participants fail to report the target rule or pattern but demonstrate a learning effect (i.e., significantly above-chance performance in judgement tests), the acquired knowledge can be considered as implicit. While verbal reports may have limitations, such as insensitivity to low-confidence knowledge or issues related to memory decay, they are still regarded as a direct and exhaustive measure of awareness (Rebuschat et al., 2015). In the current study, retrospective verbal reports were collected via a questionnaire following the experiment.

Confidence rating has also been found to be sensitive to awareness, and past studies have proposed two specific criteria for assessing it (Chan, 1992; Cheesman and Merikle, 1984; Dienes et al., 1995): the guessing criterion and the zero-correlation criterion. The guessing criterion suggests that unconscious knowledge is acquired when participants present above-chance accuracy, even if their decisions are made through guessing. The zero-correlation criterion, on the other hand, indicates that knowledge is unconscious if there is no relationship between participants’ confidence ratings and their actual accuracy. Since these criteria have been widely used and validated in previous phonological studies on implicit learning (e.g., Graham and Williams, 2018), they were employed as the primary measures of consciousness in the current study.

### 1.4 Rationale for the current study

The current study sought to investigate whether unfamiliar tone sandhi patterns existing in natural language can be learned implicitly in a word recognition situation, and whether the resultant knowledge is rule-based and generalizable. More specifically, we examined the learning effect of unfamiliar tone sandhi patterns from Tianjin Mandarin by Standard Chinese speakers from Beijing, with participants’ awareness carefully measured with retrospective verbal report and trial-by-trial confidence rating (Leung and Williams, 2006; Paciorek and Williams, 2015).

In the training phase, participants were instructed to focus on remembering the pronunciations of novel monosyllabic words and then learning the morphosyntactic rules (as manifested in word order) of disyllabic phrases in an artificial language. Meanwhile, they were exposed unknowingly to stimuli that incorporated the tone sandhi rule in Tianjin Mandarin. Subsequently, a judgement task was administered to assess whether there was a learning effect for both the explicit morphosyntactic rule and the implicit tone sandhi patterns. Significantly above-chance performance in this task would indicate a successful learning.

To examine the nature of the resultant knowledge through implicit learning, we tested two types of unseen phrases: those composed of learned monosyllabic words and those containing new artificial nouns. If the learning process had led to the acquisition of abstract and possibly rule-based knowledge, we anticipated a learning effect related to the sandhi patterns would generalize to these unseen phrases.

According to previous findings, the implicit learning effect can also be manifested in response latency, as speakers are generally more readily in perceiving sound sequences that are more permissible in their language (Chambers et al., 2010; Onishi et al., 2002; Vitevitch and Luce, 2005). Meanwhile, changes in reaction time data may further reflect the role of attention during the learning process (e.g., Schmidt, 1994, 2010). Hence, in our study, the reaction time data in the testing phase (counted as the interval between the ending of the auditory stimulus and the time of responding in each trial) in different stimuli types and conditions were also recorded and evaluated in the current study as complementary evidence of the presumable learning effect and an indicator of the role of attention.

## 2 Method

### 2.1 Participants

Twenty-seven native speakers of Beijing Mandarin (12 males and 15 females), with an average age of 21.43 (SD = 3.23), were recruited for the study. All of them were born and brought up in Beijing and primarily used Standard Chinese in their daily communication. None of the participants could speak Tianjin Mandarin or had lived with native Tianjin speakers, ensuring they had no prior knowledge of the target patterns in the artificial language. We selected Beijing Mandarin speakers as participants because they are unfamiliar with the tonal system and sandhi patterns in the Tianjin Mandarin. Additionally, by selecting a group of participants with a homogeneous dialect background, we were able to mitigate potential confounding effects from diverse dialectal tone systems on the learning of Tianjin Mandarin’s sandhi patterns. No visual, auditory, or cognitive disorders were reported from the participants. All participants were compensated for their participation, and written consent was obtained from each of them.

### 2.2 Materials

**Figure 2:**
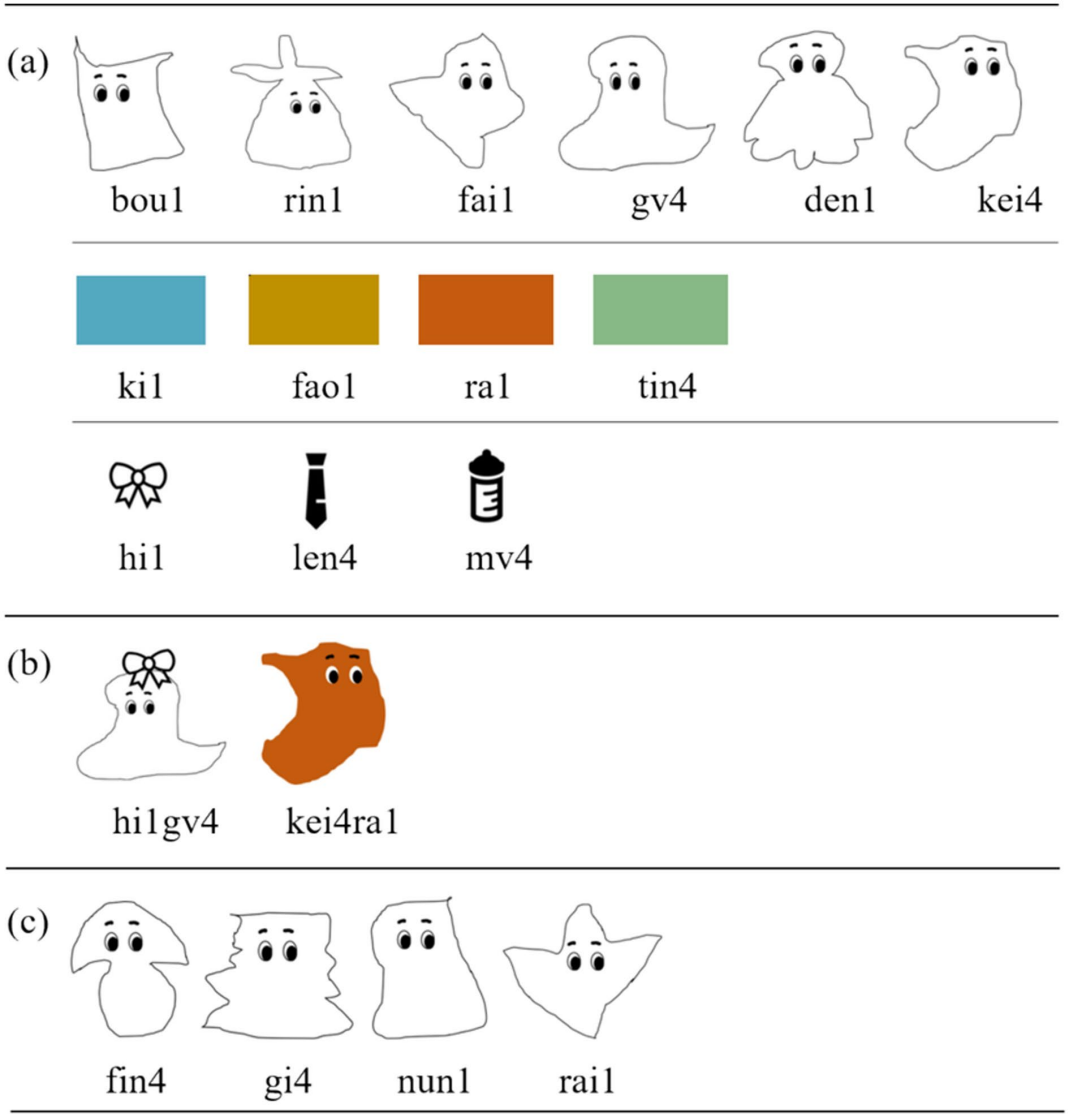
The pictures of monosyllabic words and disyllabic phrases in the artificial language. **(A)** Presents monosyllabic words used in the training session, with the bowknot, tie, and feeding bottle representing ‘female’, ‘male’, and ‘neutral’ respectively. **(B)** Provides examples of disyllabic phrases: ‘hi1 gv4’ represents ‘a female creature whose name is gv4’, where the feminine gender marker precedes the noun, while ‘kei4 ra1’ represents ‘a red creature named is kei4’, with the noun appearing before the colour words. **(C)** Introduces new monosyllabic nouns, specifically the names of four creatures, used in the second testing session.

Thirteen tone-bearing monosyllabic words were introduced in the artificial language, each corresponding to a specific picture. These words included six nouns (i.e., ‘rin1 /ɻin1/’, ‘bou1 /poʊ1/’, ‘fai1 /faɪ1/’, ‘den1 /tən1/’, ‘gv4 /ky4/’, ‘kei4 /kʰeɪ4/’), four colour words (i.e., ‘ra1 /ɻa1/’, ‘fao1 /faʊ1/’, ‘ki1 /kʰi1/’, ‘tin4 /tʰin4/’), and three gender markers (‘len4 /lən4/’ for male, ‘hi1 /xi1/’ for female, and ‘mv4 /my4/’ for neutral), as shown in Figure 2A. The numbers 1 and 4 in the *pinyin* and IPA transcription indicate T1 and T4, respectively. To ensure learnability, all these monosyllabic words were constructed in accordance to the CV(C) syllable template in Standard Chinese and Tianjin Mandarin, with a single-consonant as onset, a vowel/diphthong as rime, and an optional coda (either an alveolar nasal /n/ or a velar nasal /ŋ/; cf. Duanmu, 2007).

To avoid potential lexical interference from existing morpheme, only syllabary accidental gaps were used. This means that within each syllable, both the initial consonant (e.g., b /p/) and the rime (e.g., ou /oʊ/) were selected from the segmental inventory, but their combination did not exist in either Standard Chinese or Tianjin Mandarin (e.g., bou1 /poʊ1/; Zhang and Peng, 2013). Each monosyllabic word was assigned either T1 or T4 in Tianjin Mandarin, with the proportions of T1- and T4-bearing words across the three categories (i.e., “creatures,” “colours,” and “genders”) carefully balanced to ensure a sufficient number of disyllabic phrases in both the training and testing sessions could follow the T1/T4 + T1 sandhi rule. These monosyllabic words, along with their corresponding pictures, were used as the material for the first training session.

In the second training session, the previously introduced monosyllabic words were combined into disyllabic phrases, following specific word order rules: either gender marker + noun (e.g., ‘hi1 gv4’, denoting a female creature ‘gv4’) or noun + colour word (e.g., ‘kei4 ra1’, denoting a reddish creature ‘kei4’; see Figure 2B). This word order rule was based on French, in which the gender marker precedes the noun and the colour word follows the noun. This morphosyntactic rule was unfamiliar to the participants, which would divert their attention from the phonological aspect.

Overall, 42 potential phrases were generated. However, to balance the distribution of phrases for each word order and keep the learning load manageable, only 24 of these phrases were included in the second training session. This subset included 16 sandhi phrases (8 with T4T1 sandhi and 8 with T1T1 sandhi) and 8 non-sandhi phrases (4 with T1T4 and 4 with T4T4). Each phrase was repeated 5 times to ensure the participants were fully exposed to the morphosyntactic and the tone sandhi patterns. In total there were 120 trials (24 trials × 5 times) in the second training session.

The testing phase also consisted of two sessions. During the first session, participants were initially tested on the 24 phrases they had practiced (referred to as the Old Combinations condition, OC hereafter). Afterward, they were tested on the remaining untrained phrases selected from the 42 possible phrases (referred to as the New Combinations condition, NC hereafter). In the second session, participants were tested with new phrases constructed by new artificial nouns (Figure 2C) and learned gender markers or colour words, following the word-order rule (referred to as the New Noun condition, NN hereafter).

To examine the learning effect of the word-order and tone sandhi rules, both rule-compliant and rule-violating phrases were included. Phrases with reversed word order (i.e., “noun + gender marker,” and “colour word + noun”) were used as violators of the word-order rule. Phrases that did not show sandhi patterns when they should have (e.g., T4 in the T4 + T1 combination remained a canonical high-falling tone instead of changing according to the sandhi rule) were used as violators of the sandhi rule. Moreover, phrases where no sandhi rule should be applied (i.e., T1 + T4 and T4 + T4 phrases) served as the baseline. Altogether, six types of stimuli were formed, namely the “Correct Order-Correct Sandhi type” (CO-CS), the “Wrong Order-Correct Sandhi type” (WO-CS), the “Correct Order-No Sandhi type” (CO-NS), the “Wrong Order-No Sandhi type” (WO-NS), the “Correct Order-Wrong Sandhi type” (CO-WS), and the “Wrong Order-Wrong Sandhi type” (WO-WS; Table 1). In each type, there were 32 trials, evenly divided into OC and NC conditions with 16 trials in each, resulting in a total of 192 trials in the first testing session. For the second testing session (the NN condition), there were 16 trials for each type, resulting in a total of 96 trials.

**Table 1:** Examples for the six types of phrases used in the testing sessions.

Type	Have sandhi form?	Apply sandhi rule?	Correct word order?	Example	Correct answer
CO-CS	Yes	Yes	Yes	*rin1^s^ ra1*	TRUE
WO-CS	Yes	Yes	No	*ra1^s^ rin1*	FALSE
CO-NS	No	No	Yes	*gv4 tin4*	TRUE
WO-NS	No	No	No	*tin4 gv4*	FALSE
CO-WS	Yes	No	Yes	*rin1 ra1*	FALSE
WO-WS	Yes	No	No	*ra1 rin1*	FALSE

The superscript ‘s’ indicates the sandhi form of the tone is used.

If the explicit morphosyntactic learning has been learned, participants would accept the CO-CS, CO-NS, and CO-WS phrases as correct, as these phrases observe the word-order rule, while rejecting phrases of the WO-CS, WO-NS, and WO-WS types as incorrect due to violations of the word-order rule. On the other hand, if participants have successfully acquired the implicit sandhi rule, they would consider the CO-CS, WO-CS, WO-NS and CO-NS types to be correct, while perceiving the CO-WS and WO-WS types as unnatural or strange. In other words, only phrases that align with both the word order and tone sandhi patterns were classified as “correct” in data analysis. Among the types, CO-WS is crucial for demonstrating the potential learning effect of the implicit sandhi pattern, as it features correct word order but violates the sandhi rules.

**Figure 3:**
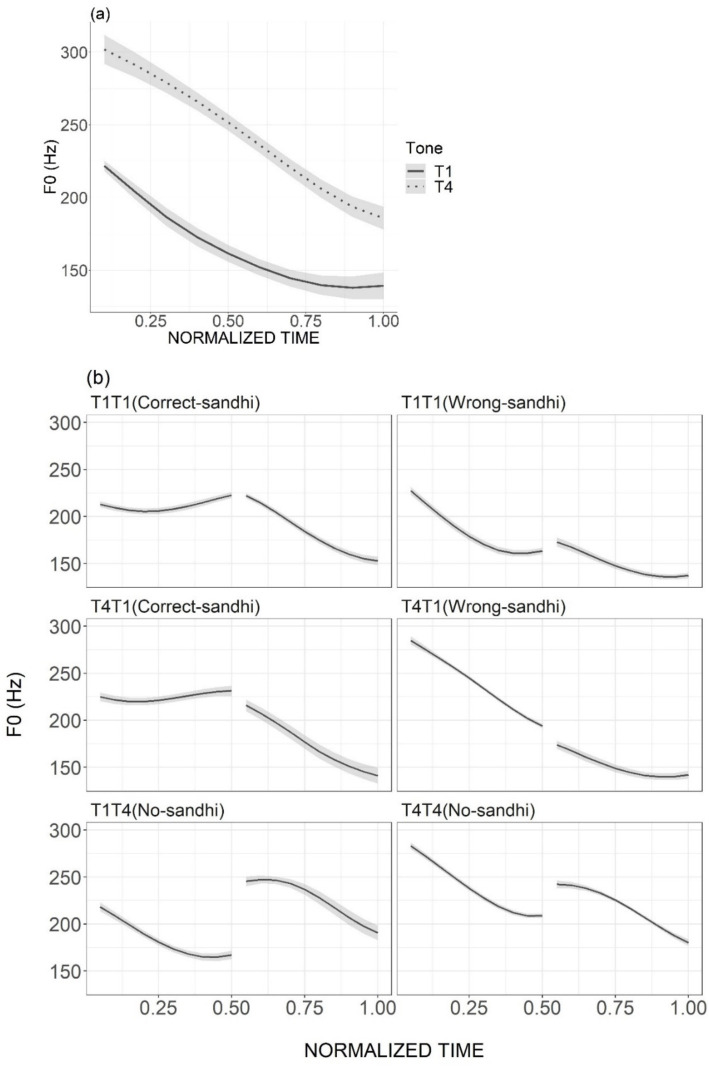
Mean f0 contours over the rime part (averaged over stimuli) of T1 and T4 in monosyllabic artificial words **(A)** and in disyllabic artificial phrases **(B)**. The ribbons represent standard errors.

In both the training and testing sessions, the auditory stimuli of the words and phrases were recorded by a female native Tianjin Mandarin speaker. For the Wrong-Sandhi phrases, the speaker was specifically instructed to ignore the sandhi rule and produce the canonical tone forms instead. The duration measurements of the syllables and f0 extraction of the tone-bearing rimes were conducted using Praat (Boersma and Weenink, 2021). To obtain the tonal f0 contours, 10 equidistant points were selected for each rime, with time-normalized f0 extraction performed using a custom-written Praat script (Arantes, 2021). Any occasional octave jumps were properly adjusted. The mean f0 contours of the monosyllabic words used in the first training session are presented in Figure 3A, with pitch height and direction matching the citation forms documented in previous studies (e.g., Li and Chen, 2016). Similarly, the mean f0 contours of disyllabic phrases (Figure 3B) used in the second training session exhibited a sandhi pattern consistent with prior research (e.g., Li and Chen, 2016; Figure 1).

The average duration of monosyllabic words carrying T4 was 346.3 ms (SD = 84.95 ms), while the average duration of monosyllabic words bearing T1 was 370.2 ms (SD = 122.67 ms). For disyllabic phrases, the average duration for phrases without sandhi was 723.50 ms (SD = 69.54 ms). The first syllable of these phrases had a mean duration of 379.06 ms (SD = 55.42 ms), and the second syllable showed a mean duration of 343.44 ms (SD = 73.28 ms). Phrases with sandhi, on the other hand, had an average duration of 689 ms (SD = 60.25 ms), with the first syllable averaging 361.50 ms (SD = 57.49 ms) and the second syllable averaging 328 ms (SD = 62.31 ms).

### 2.3 Procedure

Each participant was tested individually using E-prime 3.0 software (Psychology Software Tools, 2016) on a laptop computer in a quiet classroom. In the first training session, participants were asked to learn 13 new words in an artificial language by associating the pronunciation of each word with the corresponding picture on the screen. First, the six nouns were trained and then tested in a quiz. Subsequently, the colour words and gender markers were trained and tested in the same manner.

**Figure 4:**
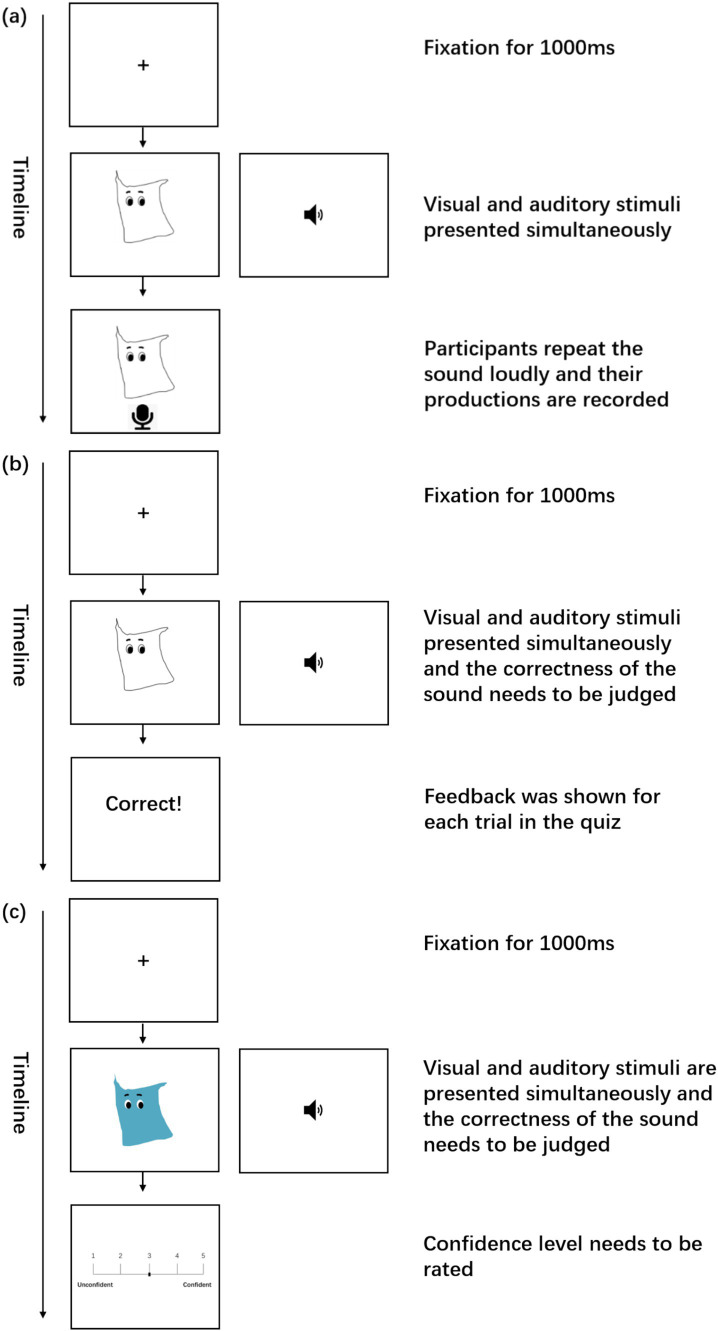
The timeline of the trial in the training phase **(A)**, the quiz **(B)**, and the testing phase **(C)**.

At the beginning of each training trial, participants were asked to gaze at a fixation cross on the screen for 1,000 ms. Following this, a picture and the auditory stimulus of an artificial word were presented simultaneously. Once the sound ended, a recording icon appeared, prompting participants to repeat the sound they had just heard aloud. A detailed timeline of a sample trial is illustrated in Figure 4A.

The six artificial nouns were presented five times, with the order randomized for each participant. Following this, a quiz (Figure 4B) was conducted to test the learning of the nouns. In the quiz, half of the trials featured correct picture-sound matches, while the other half presented incorrect matches. Participants were asked to press “j” on the keyboard for a correct match and “k” for an incorrect match. To allow participants to allocate some of their attention on the prosodic aspect of the new language, sound stimuli with tonal errors were included (e.g., T1 was produced as T4). This was important because if they did not attach any attention to tones, it would be very difficult to learn the tonal patterns in disyllabic phrases later (Leung, 2004).

To ensure proper association between pictures and their corresponding sounds, a 0.9 accuracy rate was set as a criterion. Participants who failed to meet this criterion were asked to repeat the learning process. Once they passed the quiz, participants would continue to learn colour words and gender markers with the same learning procedure.

Once all the 13 artificial words had been learned, participants proceeded to the second training session, in which they were presented with disyllabic phrases of gender marker + noun or noun + colour word combinations, and were asked to figure out the regularities in word orders. The learning procedure in this session was similar to that in the first session. A criterion of 0.8 accuracy was used to make sure the phrases had been successfully learned. Considering that the learning task in this session was more challenging than in the first session, we set a lower accuracy criterion. This adjustment was made to avoid participant fatigue from prolonged and repetitive learning, which could otherwise compromise the validity of the results. It should be noted that, the sandhi rule was properly applied in this session, that is, all T1 and T4 were pronounced as rising tones when followed by T1.

Immediately after the training, participants entered the perception testing phase. Before the test began, they took another quiz to ensure they still remembered the monosyllabic words. If their score was below 0.9, their data would be excluded from the analysis. In the testing session (Figure 4C), participants were asked to judge whether the sounds of the phrases matched the pictures on the screen as fast and accurate as possible. Similar to the training session, “j” indicated that the pronunciation sounded normal and familiar to what they had been trained on, while “k” indicated an unfamiliar or abnormal pronunciation. The key difference in the testing phase was that all 6 types of stimuli were involved. That is, the CO-WS type was also tested in this session. For certain phrases with correct word order, the tone sandhi rule may not have been applied correctly.

During this process, reaction time was recorded as the interval from the end of the auditory stimulus to the time the response was made in each trial. After each trial, the participants also required to rate their confidence level on a scale of 1 to 5, with “1” referring to “Unconfident” and “5” indicating “Confident.”

When the first testing session was complete, participants were informed that they would encounter some new creatures. With the same procedure, participants needed to judge whether the sound of the phrases containing new nouns were correct or not. After each trial, they were also asked to rate their confidence level.

After the experiment, participants filled in a post-test questionnaire, in which their language backgrounds and their awareness of the rules in the experiment were enquired. The training phase generally lasted about 20 min, while the testing phase took between 40 and 50 min. Participants were asked to take breaks between sections to prevent fatigue from affecting the experimental results.

## 3 Results

### 3.1 Results of the training phase

In the first training session, participants repeated the training session of nouns for 2.28 times on average (SD = 3.05) before achieving 0.9 accuracy in the quiz. For the colour words and gender markers, participants repeated the training session an average of 1.24 times (SD = 0.51) before reaching 0.9 accuracy in the quiz. Upon passing, participants achieved an accuracy of 0.93 (SD = 0.03) in the noun quiz and 0.94 (SD = 0.02) in the colour word and gender marker quiz. The results indicated that they successfully memorized the pronunciations of the monosyllabic words and could differentiate between T1 and T4 rather accurately.

In the second training session, all participants passed the quiz (> 0.8) after one round of training. Their mean score was 0.91 (SD = 0.05), indicating that the explicit word-order rule in trained phrases had been learned with success. Following the training, participants completed a monosyllabic word memory task, achieving a score of 0.98 in which they scored 0.98 (SD = 0.03, Min = 0.91). This suggests that before the test, all participants had effectively memorized the pronunciation of each monosyllabic word.

### 3.2 Results of the retrospective verbal reports

Based on the responses in the questionnaire, two participants reported making judgements based on a rule of tonal deviation: the first syllable took a rising tonal contour when followed by a low or mid-falling tone. These two participants were categorized as the “Aware Group.” The remaining 25 participants, who reported no tone-related issues, were classified as the “Unaware Group.”

Within the Aware Group, one participant became aware of the aforementioned rule during the second training session, while the other discovered the rule during the first testing session. In the CO-WS type, which addressed tonal knowledge, the Aware Group achieved accuracy of 0.84 in the OC condition, 0.88 in the NC condition, and 0.75 in the NN condition, all of which were well above chance level. The Aware Group demonstrated higher accuracy in judgements based on tone sandhi but at the cost of lower word order accuracy. Unlike the Unaware Group, which achieved very high accuracy rates (all above 0.9) in the five stimuli types (excluding the CO-WS type), the Aware Group only managed to achieve 0.77 accuracy.

Since the two participants were successful explicit learners of the tone sandhi rule, their results were excluded from the statistical analyses in later sections. The remaining 25 participants were placed in the Unaware Group. Sections 3.3 and 3.4 present the analyses of their performance in the testing sessions.

### 3.3 Performance in the word judgement task

**Figure 5:**
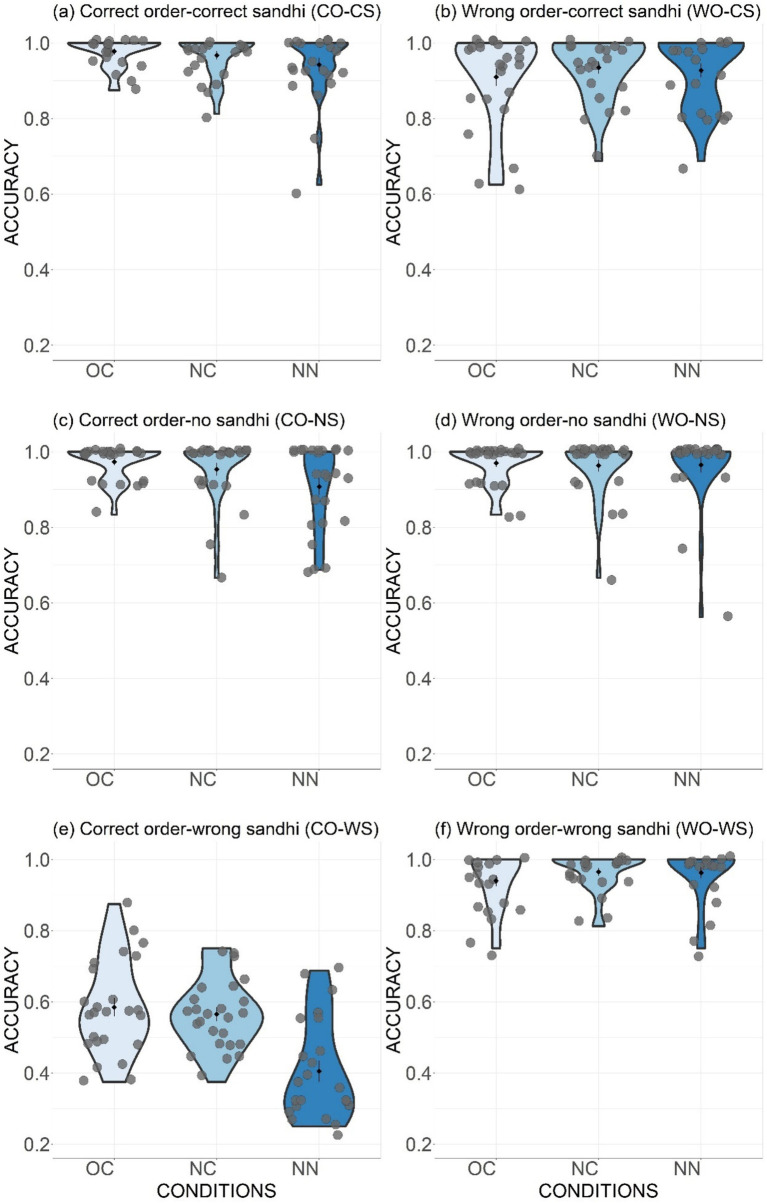
Distribution of accuracy in phrase judgement task across six types **(Panels A–F)** and three conditions (OC, NC and NN). Black diamonds in the violin plots represent type means and grey points refer to individual participant’s mean accuracy. The error bars stand for standard errors.

The mean acceptance rates across the six stimuli types and three conditions for the “Unaware Group” are presented in Figure 5. The results were analyzed using generalized mixed-effects model in R (R Core Team, 2020) with the lme4 package (Bates et al., 2015), starting with a full model. Model comparisons revealed significant effects of the following fixed factors: Type of stimuli (i.e., CO-CS, WO-CS, CO-NS, WO-NS, CO-WS, WO-WS), Condition [i.e., OC (Old combination), NC (New combination), and NN (New nouns)], the interaction of Type and Condition, as well as Word Order (Gender marker + Noun and Noun + Colour word). By-item and by-participant intercepts were included as random effects [glmer formula: Accuracy ~ Type * Condition + Word Order +(1|Item) + (1|Participant)]. The χ^2^ and corresponding *p*-values for the fixed effects were obtained from likelihood ratio tests. To further compare the differences among individual levels of the fixed effects, post-hoc comparisons were conducted using the *glht* function in the Multcomp package (Hothorn et al., 2008) with Bonferroni adjustment in R. For reaction time, the raw RT data was logarithmically transformed to achieve better normalcy and analyzed in a similar linear mixed effect model [lmer formula: LogRT ~ Type * Condition + Word Order + (1|Item) + (1|Participant)].

The results of the accuracy rate model (Table 2) suggested that the effects of Stimuli Type, Stimuli Condition, their interaction, and the effect of Word Order were all significant. Given that the word order of gender word + noun aligns with word order pattern in Standard Mandarin, it is unsurprising that participants performed significantly better in judging gender word + noun phrases (accuracy of 0.88) compared to noun + colour word phrases (accuracy of 0.86) [*χ^2^*(1) = 9.3, *p* < 0.001]. The acceptance rate results of each stimuli type are demonstrated first in the following section, followed by comparisons across different stimuli types to illustrate the participants’ learning performance.

**Table 2:** Summary of the mixed-effect models for response accuracy and reaction time.

	Acceptance			RT		
Fixed effects	Df	*χ^2^*	*p*	Df	*χ^2^*	*p*
Condition	2	25.9	< 0.001	2	712.61	< 0.001
Type	5	352.51	< 0.001	5	226.87	< 0.001
Condition × Type	10	30.57	< 0.001	10	26.23	< 0.01
Word Order	1	9.3	< 0.01	1	405.15	< 0.001

As introduced in section 2.3, the Correct Order-Correct Sandhi (CO-CS) type consisted of stimuli in which the sandhi rule should and had been properly applied (i.e., tonal combinations of T1T1 and T4T1). For this type (Figure 5A), participants demonstrated very high accuracy in endorsing the stimuli as proper phrases across all conditions: OC (M = 0.98), NC (M = 0.97), and NN (M = 0.95). The only significant difference was observed between the OC and NN conditions (β = 0.98, z = 2.46, *p* < 0.05), indicating a slight decrease of performance when dealing with unfamiliar nouns.

Regarding the stimuli of Wrong Order-Correct Sandhi (WO-CS) type (Figure 5B), the explicit word-order rule was violated, despite the correct application of the sandhi rule on the T1 in the preceding syllable. Overall, participants effectively rejected these incorrectly ordered phrases, with similar performance across conditions (OC: M = 0.91; NC: M = 0.94; NN: M = 0.93).

The acceptability of the Correct Order-Non-Sandhi (CO-NS) stimuli can be judged using the explicit word-order rule, since the sandhi rule does not apply in this type (involving the tonal combinations T1T4 and T4T4). Across the three conditions (OC: M = 0.97; NC: M = 0.95; NN: M = 0.91; Figure 5C), participants showed significantly higher accuracy in the OC condition compared to the NN condition (β = 1.33, z = 3.34, *p* < 0.01), suggesting a better performance with phrases that had been learned. The Wrong Order-Non-Sandhi (WO-NS) condition could also be judged according to the word-order rule. Similar to the previous type, participants could easily and accurately reject the incorrectly ordered phrases of this type (OC: M = 0.97; NC: M = 0.96; NN: M = 0.97; Figure 5D). The accuracy rates showed no significant differences across the three conditions (all *p* values >0.05).

To determine the learning effect of the sandhi rule, the performance in the Correct Order-Wrong Sandhi type (CO-WS) was critical. If there is a learning effect, these stimuli, which violated the sandhi rule (since the preceding tones in T1T1 and T4T1 combinations were not changed to rising tones), should be judged as unacceptable, despite their correct word order. In the OC condition (Figure 5E), 0.59 (SD = 0.13) of the stimuli were correctly rejected, significantly above the chance level (0.5), as indicated by a generalized mixed model with the intercept significantly above 0 [glmer formula: Accuracy ~1 + (1|Participant) + (1|Item); β = 0.35, z = 3.24, *p* < 0.01]. This result indicates a learning effect, which generalized to new combinations, as shown by significantly higher accuracy in the NC condition (M = 0.57, SD = 0.1) compared to chance level (β = 0.26, z = 2.59, *p* < 0.01). However, the accuracy in the NN condition (M = 0.39, SD = 0.15) fell below the chance level (β = −0.47, z = −3.75, *p* < 0.001), indicating that when judging the acceptability of phrases with new nouns, participants did not rely on the sandhi rule as a critical criterion.

Stimuli from the Wrong Order-Wrong Sandhi type violated both word-order and sandhi rules and were corrected rejected as unacceptable by the participants in all three conditions with very high accuracy rates (OC: M = 0.94; NC: M = 0.97; NN: M = 0.96; Figure 5F). There were no statistically significant differences in accuracy across the conditions (all *p* values >0.05).

For the types of CO-NS and WO-NS (Figure 5C vs. Figure 5d), only the word-order rule needs to be considered when making judgements, as the sandhi rule does not apply to the tonal combinations in these types (i.e., T1 + T4 and T4 + T4). The results showed that participants could correctly endorse the correct stimuli (Figure 5C) and reject the wrong ones (Figure 5D) with very high accuracy rates. *Post hoc* results revealed no significant difference between these two types across all three conditions (all *p* values >0.05). This indicates a strong grasp of the explicit word-order rule when there was no interfere from the sandhi aspect.

For the CO-CS and WO-CS types (Figure 5A vs. Figure 5B), judgements could also be made by considering only the word-order rule, as the sandhi rule had been properly applied in both types. Following the logic of the CO-NS vs. WO-NS comparison, we would expect similar comparable accuracies between the CO-CS and WO-CS types. However, the results exhibited a significant decrement in accuracy for the WO-CS type compared to the CO-CS type in the OC condition (β = 1.47, z = 3.88, *p* < 0.01). This suggests that the sandhi pattern might have been adopted as an additional criterion, leading to a higher degree of endorsement for stimuli with incorrect word order but correct sandhi pattern.

The role of the sandhi rule as a criterion of judgement is further supported by the comparison of the WO-CS and WO-WS types (Figure 5B vs. Figure 5F). Participants were able to correctly reject stimuli that violated both rules, but they might have been “lured” to accept the stimuli with incorrect word order but correct sandhi patterns as valid forms, as reflected by the generally lower accuracy rates for the WO-CS type.

**Figure 6:**
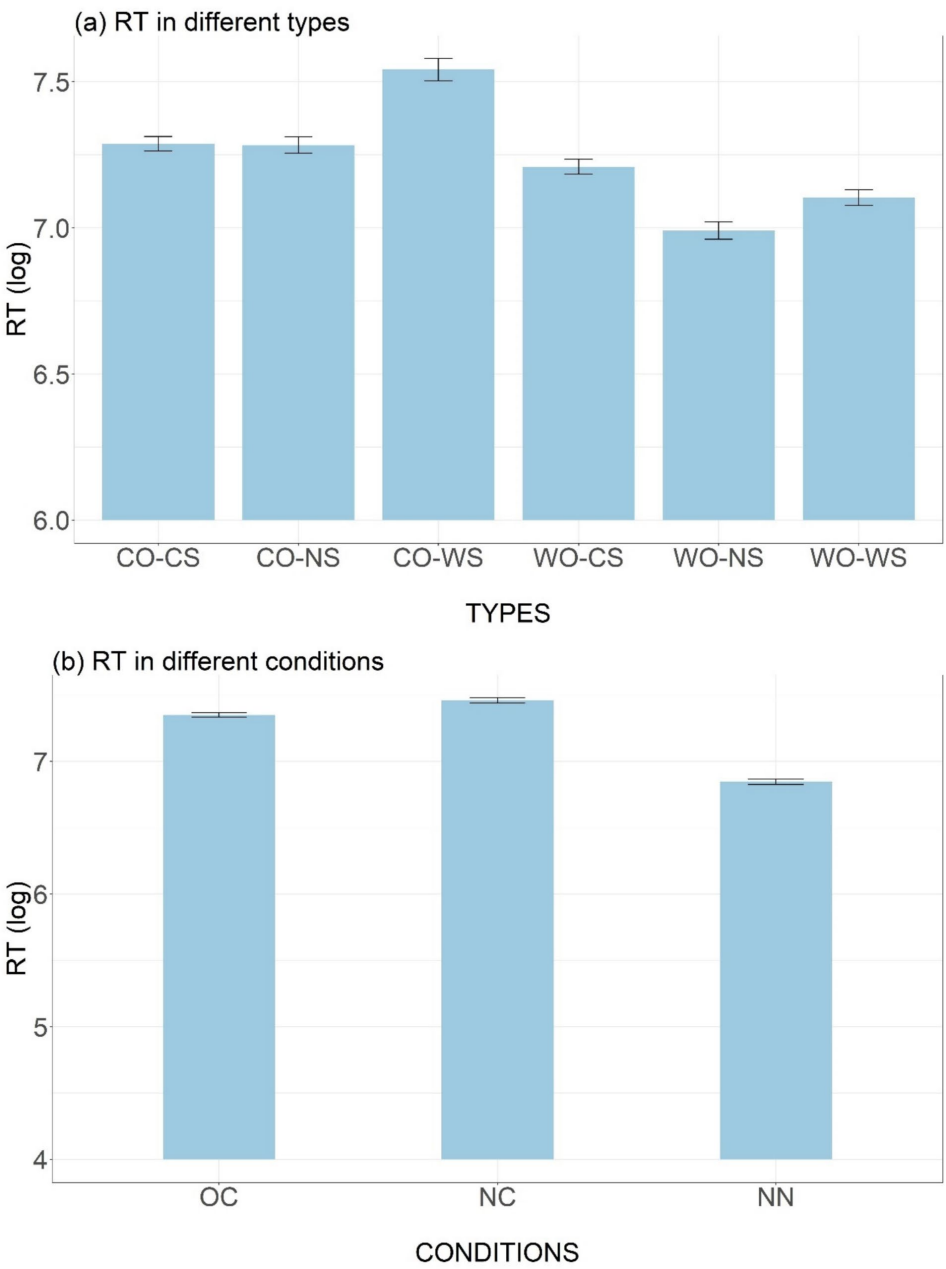
Mean RT results across participants for six types **(A)** and three conditions **(B)**. Error bars represent standard errors.

In terms of reaction time, it can be observed that the six types generally fell into two subgroups, with the types with correct word order consuming more time compared to those with incorrect word order (Figure 6A). Such contrast can be attributed to the intrinsic differences between the stimulus types, as the wrong-order stimuli were not consisted with the expected correct phrase from the first word (syllable), while for the correct-order types, participants needed to wait until the second word (syllable) in order to make a judgement. According to the *post hoc* results, all contrasts between members from these subgroups were statistically significant (all *p* values <0.05).

For the stimuli types with correct order (Figure 6A), participants responded more rapidly to legal types which obeyed the sandhi rule (CO-CS and CO-NS) compared to the illegal type that violated the sandhi rule (CO-WS; CO-CS vs. CO-WS: β = −0.09, z = −3.17, *p* < 0.05; CO-NS vs. CO-WS: β = −0.16, z = −5.21, *p* < 0.001). This legality advantage in RT presumably reflects the learning effect of the sandhi pattern, which echoed the findings from the accuracy data. For the wrong-order types, participants responded significantly more quickly to the WO-NS type compared to the WO-CS (β = 0.20, z = 5.46, *p* < 0.001) and WO-WS types (β = 0.16, z = 5.21, *p* < 0.001). This may indicate that participants noticed the tonal variations, as both correct and incorrect applications of the sandhi rules could draw their attention and led to hesitation, which was reflected in their reaction times.

Figure 6B shows that participants were more readily to respond for learned phrases (the OC condition) than phrases with new combination of old words (the NC condition), as indexed by significant difference in RT (β = 0.11, z = 4.81, *p* < 0.001). The RT was shortest in the NN condition, as participants were directly presented with the pronunciation of the new noun, eliminating the need to retrieve it from memory.

### 3.4 Results of the confidence rating task

**Figure 7:**
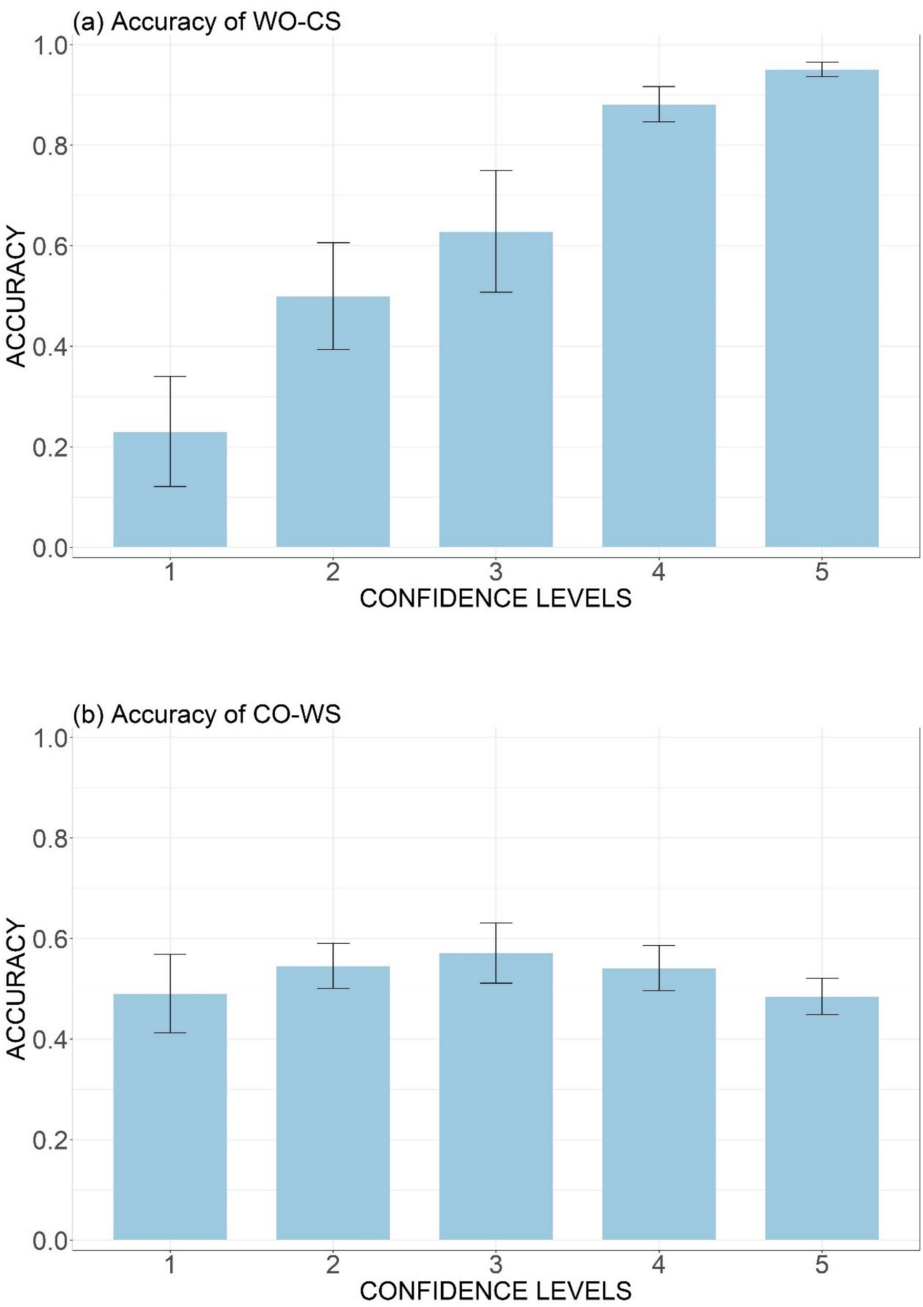
Mean accuracy rates across participants at different confidence levels (1–5) for the WO-CS type **(A)** and the CO-WS type **(B)**.

As shown in Figure 7A, there is a clear positive correlation between accuracy and participants’ confidence rating for the Wrong Order-Correct Sandhi stimuli. At the lowest confidence level, the accuracy was only 0.23, indicating that participants may have been aware of their erroneous decision when rating their confidence. The high accuracy (0.95) at the highest confidence level (level 5) also suggests a good command of the order rule in an explicit manner.

However, when the word order remained intact but the sandhi rule was violated (the CO-WS type), as shown in Figure 7B, there was no clear correlation between accuracy and participants’ confidence levels. The accuracy rate exceeded chance level when confidence rating was 2 (0.55), 3 (0.57), 4 (0.54) and was slightly below chance level (0.49) when confidence rating was 5. These evidences collectively exhibited that participants were unaware of the specific content of the sandhi rule. Consequently, their above-chance performances were likely based on intuition.

**Figure 8:**
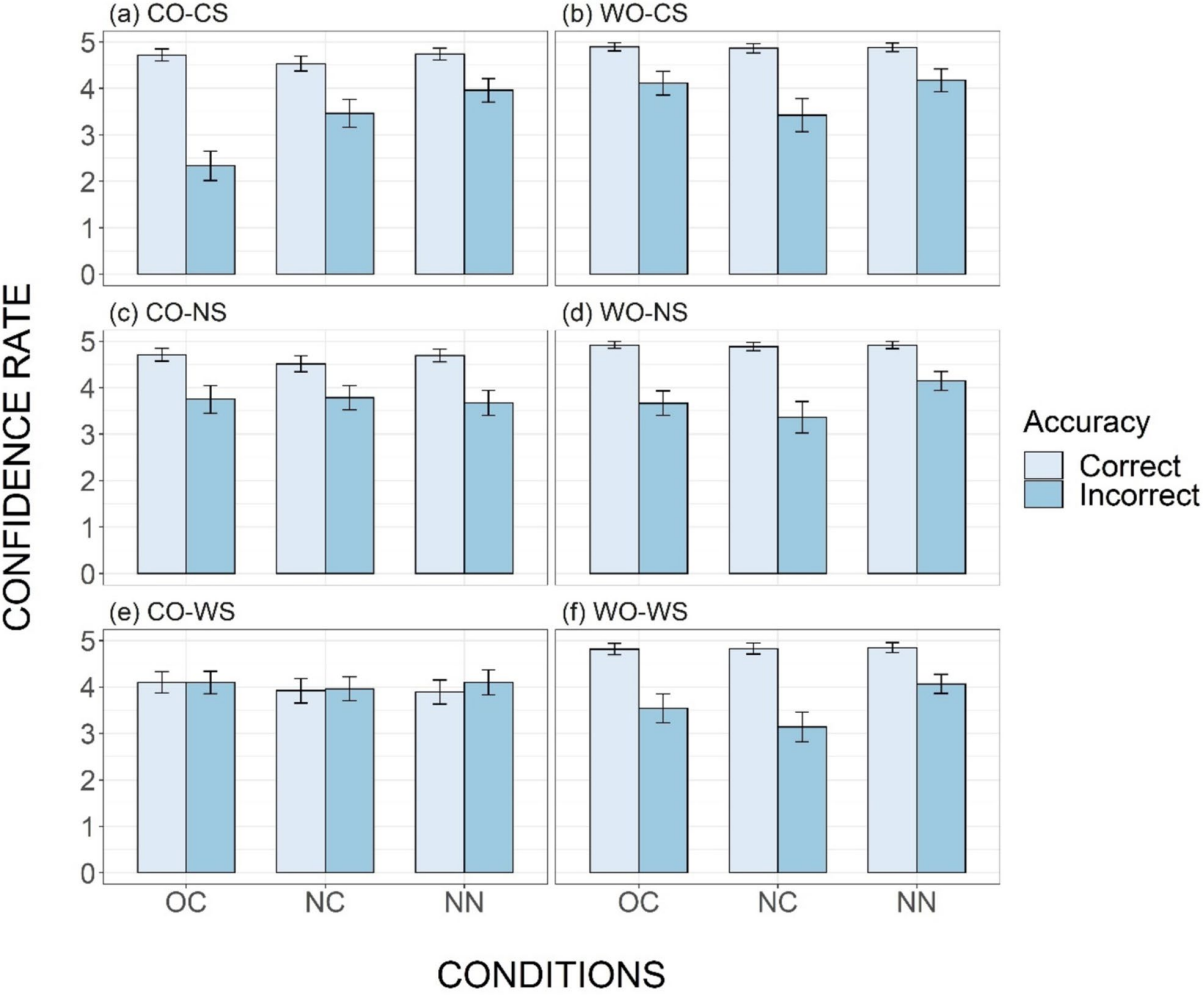
Mean confidence ratings for correct and incorrect judgements, across six types **(Panels A–F)** and three conditions (OC, NC, and NN). The error bars indicate standard errors.

To further illustrate the difference in participants’ level of confidences between accurate and inaccurate judgements, confidence rating data was analyzed using linear mixed-effects models with the lme4 package in R. For each stimulus type, a model was built with Condition (OC, NC, NN), Accuracy (correct or incorrect) and their interaction as fixed effects [Confidence ~ Accuracy*Condition + (1|Participant) + (1|Item)]. For all six models, by-participant and by-item intercepts were included as random effects. Confidence rates grouped by accuracy were plotted across conditions and stimulus types in Figures 8A–F.

The results demonstrated that the effect of Accuracy was significant for all stimuli types, except the CO-WS type (Table 3; Figure 8). For most types, the confidence rating was much higher for correct judgements than for incorrect ones, indicating participants’ conscious use of the word-order rule. Additionally, the difference in confidence ratings between correct and incorrect responses was larger in the OC condition than in the NN condition for the CO-CS, WO-CS, WO-NS, and WO-WS types, as indicated by significant interaction between Accuracy and Condition for these types (Figures 8A,B,D,F; Table 3). This suggests that participants were more aware of the accuracy of their judgements in familiar phrases than in unfamiliar ones.

**Table 3:** Summary of linear mixed-effects models for confidence rates in six types.

Fixed effects	Df	*χ^2^*	*p*	*χ^2^*	*p*	*χ^2^*	*p*
		CO-CS		WO-CS		CO-NS	
Accuracy	1	88.93	<0.001	135.84	<0.001	51.64	<0.001
Condition	2	11.47	<0.01	2.08	n.s.	12.54	<0.01
Accuracy:Condition	2	45.87	<0.001	24.10	<0.001	0.56	n.s.
		WO-NS		CO-WS		WO-WS	
Accuracy	1	134.46	<0.001	0.21	n.s.	140.6	<0.001
Condition	2	2.03	n.s.	2.69	n.s.	3.19	n.s.
Accuracy:Condition	2	16.45	<0.001	1.31	n.s.	20.17	<0.001

The effects of Accuracy, Condition, and their interaction, on the other hand, were not significant for the CO-WS type (Table 3; Figure 8E). For this type, confidence levels for inaccurate and accurate responses were similar across all conditions (OC: 4.09 and 4.10; NC: 3.96 and 3.92; NN: 4.10 and 3.89). Participants did not exhibit higher confidence for correct answers, and the lack of correlation between confidence levels and accuracy meets the zero-correlation criterion, indicating implicit acquisition of tone sandhi pattern knowledge.

## 4 Discussion

### 4.1 Learning effect of the unfamiliar tone sandhi patterns

In this study, we explored whether Standard Mandarin speakers could implicitly learn Tianjin Mandarin tone sandhi patterns and whether the resultant knowledge was potentially rule-based and generalizable. The results revealed that for the explicit word-order rule which the participants were instructed to learn, they could accept the legal phrases and reject the rule-violated illegal phrases with very high accuracy (the CO-CS and WO-CS types).

Such learning effect also extended to unseen phrases constructed from learned words and unseen phrases containing new nouns, indicating successful acquisition of the word order rule. For the implicit tone sandhi patterns (the CO-WS type), a learning effect was also observed, as participants’ performance was significantly above chance in judging learned phrases. This learning effect also held for unseen phrases constructed from learned words, but did not generalize to new phrases containing new nouns.

With respect to the nature of the current resultant knowledge, the findings suggested that the learning is not solely based on individual instances. In the training phase, participants did not simply memorize the disyllabic phrases as particular chunks. The significantly above-chance performance for unseen combinations of old nouns, gender markers and colour words indicated certain extent of generalization beyond learned instances had occurred, likely through automatic and unconscious pattern-detection mechanisms.

However, the available data did not fully support the rule-based learning theories, as the learning effect disappeared when phrases consisting of new nouns were encountered (reflected in the weaker performance in the NN condition of the CO-WS type). Such limited generalization suggests that tone sandhi learning is not an all-or-none process. Instead, knowledge may emerge gradually through the accumulation of repeated experience in perceiving individual instances. This pattern generally aligns with the concept of categorical learning as explored by the research of Erickson and Kruschke’s (1998). In their study, participants were asked to classify items that followed a simple rule. Two experiments examined how individuals extrapolated beyond the training range and how the frequency of instances influenced generalization. The categorization behavior was effectively described by a model that captured the interplay between rule-based and exemplar-based representations. This model essentially highlights that both rule induction and exemplar encoding contribute to an integrated representation.

Another notable point is the relatively less desirable learning effect in the current study compared to previous findings on word stress learning, where non-native word stress patterns can be learned rapidly and successfully transferred to new items (e.g., Graham and Williams, 2018; Chan and Leung, 2018). We argue that this difference may be partially due to the differential learning conditions: in the current experiment, the sandhi knowledge was acquired in a word recognition task, which required mapping tonal patterns to specific meaning. In contrast, most previous lexical stress studies did not involve semantic processing. That is, the difficulty and complexity of the target language pattern, as well as the learning paradigm, may influence the learning effect of unconscious knowledge (Fu et al., 2008).

Lexical knowledge plays a crucial role in this study, as the implicit learning of the sandhi rule is dependent on the nouns that have already been learned. Consequently, the interplay between word learning and the implicit learning of tone sandhi rules becomes a fascinating area for investigation. Is lexical knowledge a prerequisite for triggering the learning of prosodic rules like tone sandhi? To what extent is lexical knowledge necessary to initiate the acquisition of such prosodic rules? These issues are interesting topics for further research.

### 4.2 The assessment of participants’ awareness

In terms of participants’ consciousness, for the type related to the learning of the word-order rule (the WO-CS type), the clear positive correlation between confidence rate and accuracy suggests participants were aware of the criterion for judgement. On the other hand, the lack of such a correlation between confidence level and participants’ performance implies that knowledge of sandhi patterns was acquired in an implicit manner. This result was further supported by the lack of relationship in the confidence levels for accurate and inaccurate answers in the sandhi-related CO-WS type, which satisfied the zero-correlation criterion, a commonly used criterion to confirm the implicitness of the learning process.

However, a close examination of the RT and confidence rating data revealed that, although the sandhi pattern was acquired unconsciously, attention plays an essential role in facilitating the learning. Longer RT was observed in the sandhi-violating CO-WS type compared to the sandhi-following CO-CS and CO-NS types. This difference may suggest that after exposure to the new language, participants had noticed the “weirdness” in the illegal stimuli and started to allocate part of their attentional resources to the tonal aspects of the new language, causing hesitation in their decisions. Such awareness at the level of noticing was also evident from the longer RT for the WO-CS and WO-WS types compared to the WO-NS type, as well as the overall high confidence rate (around 4 out of a 5-point scale) for the sandhi-related type (CO-WS). This indicates that participants had noticed the tonal variations but had not acquired structural conscious knowledge (knowledge of the structural rules of the training items). Their state of their knowledge therefore can be described as per Norman et al. (2007, p.833), “a situation in which a behaviour is driven in a flexible manner by consciously accessible feelings, but where there is no conscious access to the antecedents of those feelings.”

Such results are also in line with the “noticing hypothesis” (Robinson, 1995; Schmidt, 1994, 2010), which states that only when people notice certain aspects of linguistic knowledge can they transform the input into the intake. Schmidt also suggested that “awareness at the level of noticing” should be distinguished from “awareness at the level of understanding” (1994, p.213). That is, to encode and store instances of the new language in memory, it may be necessary for the participants to notice the specific aspects in the learning materials, but at the same time, they could still be unaware at the level of understanding, extracting regularities or rules across instances in an implicit manner (Rosa and Leow, 2004; Williams, 2009). For example, Jiang et al. (2012) indicated that tonal symmetry in Tang poetry could be acquired as structural unconscious knowledge through implicit learning.

It has also been argued that the awareness needs not to be highly saliant or deeply rooted, as Ögeyik (2018) and Ortega (2009) have noted that our brain can register new information with only fleeting awareness, even in the absence of understanding how the elements function. Such shifts in attention allocation have generally been reported as a crucial mechanism in L2 speech acquisition (e.g., Francis and Nusbaum, 2002; Zou et al., 2017).

### 4.3 Limitations and further implications of the current findings

Regarding the awareness of sandhi rules, while the questionnaire and confidence rating results suggest that participants in the Unaware Group did not realize the structural sandhi knowledge, the reaction time data indicate that this sandhi knowledge was learned with awareness at the level of noticing. We also acknowledge the possibility that some participants might have become aware of the tonal patterns in the stimuli but failed to report this awareness in the questionnaire. This could be due to the difficulty in identifying and articulating the underlying rule accurately after limited exposure to the stimuli. Therefore, to avoid this potential bias, more rigorous measures are needed to ensure implicit learning in future studies.

The present study revealed a miniature of tone reassignment in dialect adaptation and acquisition. Previously, such reassignment has been addressed in consonant allophones (e.g., German et al., 2013; Tagliamonte and Molfenter, 2007). For instance, German and colleagues (ibid.) found that American English speakers could produce the allophones of /t/ and /r/ in the fashion of Glawegian English after imitating the Glawegian speakers’ words in a 20-min training session. The exposures were automatic and unconscious, and the learning effect was retained for at least a week. Based on the current results, it can be inferred that such dialect adaptation may not be limited to segments but also exist in tones. Subsequently, the questions about how tone sandhi patterns can be learned in real-life dialect adaptation situations and whether the observed learning effects extend to speech production are likely to become interesting and significant topics for future research.

## 5 Conclusion

The current study investigated whether Standard Mandarin speakers from Beijing could learn the tone sandhi patterns in Tianjin Mandarin in an artificial language acquisition setting. The results suggest a learning effect of tone sandhi patterns, which can be generalized to the novel phrases with old words, but cannot be transferred to new phrases containing nouns that have not been encountered before. Such limited generalization suggests the extraction of regularities in tonal patterns, but the learning is not fully rule-based. Moreover, the lack of correlation between participants’ accuracy and their confidence rates in the sandhi-relevant stimuli type suggests that the sandhi knowledge was learned unconsciously. The incongruence between the implicit tone sandhi rule and the explicit morphosyntactic rules caused hesitations in participants, manifested by longer reaction times. This suggests that although participants did not explicitly figure out the structural knowledge of sandhi patterns, they noticed the variations in tonal patterns and allocated some attentional resources to the tonal aspect in the learning process. In other words, the current study invites the conclusion that tone sandhi knowledge can be learned with some extent of potentially rule-related abstraction and with awareness at the level of noticing. Whether there is a similar learning effect for tone sandhi patterns in more natural dialect adaptation circumstances, and whether such implicit learning also occurs in speech production would be worthwhile avenues for further exploration.

## Data Availability

The raw data supporting the conclusions of this article will be made available by the authors, without undue reservation.
